# The sense-of-coherence predicts health-related quality of life and emotional distress but not disability in Parkinson’s disease

**DOI:** 10.1186/s12883-014-0193-0

**Published:** 2014-10-10

**Authors:** Annalisa Gison, Federica Rizza, Stefano Bonassi, Valentina Dall’Armi, Stefania Lisi, Salvatore Giaquinto

**Affiliations:** Department of Neurological Rehabilitation, IRCCS San Raffaele Pisana, Rome, 00163 Italy; Unit of Clinical and Molecular Epidemiology, IRCCS San Raffaele Pisana, Rome, 00163 Italy

**Keywords:** Sense-of-coherence, Depression, Parkinson’s disease, Quality of life

## Abstract

**Background:**

Personality traits are deemed important in many fields of Medicine. The present study aimed at evaluating i) the presence of Sense-of-Coherence (SOC) in patients suffering from Parkinson’s Disease (PD) in comparison with an age-matched general control population, ii) the influence of SOC on health-related variables, such as depression and anxiety, quality of life (Qol), and activities of daily living (ADL).

**Methods:**

SOC was measured in 50 PD patients and in 50 matched controls enrolled in cross-sectional study. The other clinical measures included: Mini Mental State Examination (MMSE), Movement Disorder Society revision of the Unified Parkinson's Disease Rating Scale (MDS-UPDRS), Well-being Index (WHO-5), Hospital Anxiety and Depression Scale (HADS) and the Barthel Index of ADL (BI). Data were analysed with univariate statistics and loglinear adjusted regression models.

**Results:**

No difference emerged between PD and controls on socio-demographic and SOC. A statistically significant positive correlation was found between SOC and Qol (0.40, p < 0.004) and a negative significant correlation between SOC and emotional distress (−0.37, p < 0.008). The multivariate regression analysis confirmed the negative effect of SOC on total emotional distress (−3%, p = 0.01) and positive effect on Qol (2%, p = 0.01). SOC and BI were uncorrelated.

**Conclusions:**

SOC is predictive of QoL and emotional distress in PD, whereas no evidence of a predictive effect for disability could be found. These results support only partially, the Salutogenic Theory in PD, i.e. a strong SOC positively influences psychosocial health, but does not influence physical health.

## Background

Sense of coherence (SOC) is a widely applied concept in Medicine, describing person’s orientation and internal strength. This parameter can be included in a theoretical model that may explain successful coping with stressful events of life [1–3]. In such a way SOC can be considered as a psychological factor that predicts good health and positive adjustment. SOC has a “salutogenetic orientation” that is opposite to the well-known concept of “pathogenetic orientation”, i.e. the knowledge of the mechanism explaining origin and development of a disease. The SOC scale is a widely used instrument in Medicine as well as in other research areas. High SOC values indicate successful coping with life stressors. The SOC scale consists of three factors: comprehensibility (SOC-C), manageability (SOC-Ma) and meaningfulness (SOC-Me). Comprehensibility is the belief that things are predictable and happen in an orderly way. Understanding the life events and predicting what may happen in the future let a person exploit the resources required for coping. Manageability is the opinion of a person about the proper means for controlling and solving the new problems. Meaningfulness is the belief that coping makes sense is desirable.

The possible indications of ability to handle stress explain the implementation of SOC in the field of disabling diseases. In particular, SOC was studied in Parkinson’s Disease (PD). In a 1-year interval a pronounced decrease in the SOC scale was found to indicate a decreased ability to handle stress and the useful implementation of the scale in nursing care [4]. Compared to persons suffering from other non-neurological diseases, PD patients had a lower SOC score and this can be interpreted as a less positive attitude in handling stress [5].

Limited evidence is available in the Literature on the role of SOC in patients undergoing rehabilitation programs for neurodegenerative diseases such as PD. The main objectives of the present investigation are: i) to verify the difference of SOC between PD and healthy controls; ii) to evaluate the influence of SOC on health-related variables, such as Quality of Life (WHO-5, QoL questionnaire), Emotional Distress (HADS, Hospital Anxiety and Depression Scale), Activities of Daily Living (BI, Barthel Index) and illness severity in PD participants (UPDRS-Unified Parkinson’s Disease Rating Scale).

## Methods

### Participants

Seventy-five consecutive patients with diagnosis of idiopathic PD were referred by the General Practitioners (GPs) of our health district in the period 2012–2013.

*Inclusion criteria were:* confirmed diagnosis of idiopathic PD. Hoehn and Yahr’s [6,7] stages: 1, 2 and 3. Education over 5 years. *Exclusion criteria were:* parkinsonism. Cognitive Impairment. Lack of Informed Consent. High comorbidity (for example severe cardiopathy or pneumopathy).

At the end of the screening procedures, which are described below, twenty-five patients did not meet the inclusion criteria and were not included. Thus, the study consisted of fifty participants (Table 1). They were at stages 1, 2 or 3, based on the HY scale. The HY stages range from 1 to 5 and rate the stage of disease. The mean duration of illness was 6.5 years (sd 2.1). All participants lived at home with spouse or family. Eighty-one percent of them were retired. Participants had all been receiving optimized Levodopa/Dopa Decarboxilase Inhibitor therapy and/or Dopamine Agonists and were tested while on medications with optimal effects. Fifty age-matched healthy subjects were randomly selected from the district GPs’ files. Age and education of the two groups were equivalent.

**Table 1:** **Descriptive analysis of the study population (WHO-5 = Well-being index; HADS = Hospital anxiety and depression; UPDRS = Unified Parkinson’s disease rating scale; SOC = Sense of coherence)**

**Socio-demographic, clinical and psychological variables**	**Control group (n = 50)**	**Parkinson's disease group (n = 50)**	**p-value**
**Sex (n(%))**			
*Female*	22 (44%)	19 (38%)	0.54^a^
*Male*	28 (56%)	31 (62%)	
**Age (mean ± sd)**	67.34 ± 6.15	67.1 ± 9.92	0.88^b^
**Education**			
**Primary school (n(%))** *(<8 years)*	5 (10%)	13 (26%)	0.14^a^
**Secondary school (n(%))** *(8 years)*	13 (26%)	9 (18%)	
**High school/college (n(%))** *(8–13 years)*	19 (38%)	20 (40%)	
**University (n(%))** *(>13 years)*	13 (26%)	8 (16%)	
**SOC (mean ± sd)**	56.36 ± 6.77	53.84 ± 7.36	0.08^b^
**WHO-5 (mean ± sd)**		51.68 ± 19.26	-
**HADS (mean ± sd)**		14.68 ± 6.23	-
**HADS (mean ± sd) -** *anxiety*		7.98 ± 3.98	-
**HADS (mean ± sd) -** *depression*		6.86 ± 3.57	-
**UPDRS (mean ± sd)**		46.18 ± 7.50	-
**Barthel index (mean ± sd)**		56.84 ± 11.59	-

^a^ *Chi-square test,* ^b^ *Student's t-test. Statistical Significance: p < 0.05 (in bold).*

The study was approved by the Ethical committee of the IRCCS San Raffaele Pisana and an informed consent was signed by al participants.

### Measurements

*Mini Mental State Examination – MMSE* [7]. MMSE consists of questions on Orientation, Registration, Attention and Calculation, Recall, Language, Copying. MMSE is considered a screening test for cognitive impairment. The maximum score is 30 points. Values below 24 indicate mental deterioration.

*Cumulative Illness Rating Scale* [8]. The CIRS scale identifies 14 items, corresponding to different systems. Each system is scored as follows: 1 = none - no impairment to the specific organ/system; 2 = mild - impairment does not interfere with normal activity, treatment may or may not be required, prognosis is excellent; 3 = moderate - impairment interferes with normal activity, treatment is needed, prognosis is good; 4 = severe - impairment is disabling, treatment is urgently needed, prognosis is guarded; 5 = extremely severe - impairment is life threatening, treatment is urgent or of no avail, prognosis is not good. Participants with scores over 3 in any item were not admitted.

#### Movement disorder society-sponsored revision of the unified Parkinson's disease

*Rating Scale– MDS-UPDRS* [9]. The MDS-UPDRS retains the UPDRS structure of four parts with a total summed score, but the parts have been modified to provide a section that integrates nonmotor elements of PD: I – Nonmotor Experiences of Daily Living; II - Motor Experiences of Daily Living; III - Motor Examination; IV - Motor Complications. All items have five response options with uniform anchors of 0 = normal, 1 = slight, 2 = mild, 3 = moderate, and 4 = severe.

*Sense of Coherence Scale* [2]. The 13-item SOC scale measures comprehensibility (5 items), manageability (4 items), and meaningfulness (4 items). Each item in the 13-item SOC scale has seven graded (Likert-type) response scale, which is summed up and the total scores can range from 13 (low SOC) to the maximum of 91 (highest possible SOC). A validated Italian version was applied in the present study [10].

*WHO-5 Well-being Index* [11]. The scale is derived from a larger rating scale developed for a WHO project on QoL. The original items were reduced to five items (WHO-5), which covered positive mood (good spirits, relaxation), vitality (being active and waking up fresh and rested), and general interests (being interested in things). The short form is more suitable for people with a reduced endurance. The scale has been successfully applied to a PD population with Cronbach's alpha = 0.83 [12]. The Italian version [13] was applied.

*Hospital Anxiety and Depression Scale, HADS* [14]. The HADS is a self-assessment scale to measure both anxiety and depression. Higher scores mean higher psychological distress. The cut-off scores are fixed at 5 for the partial scores and at 10 for the total score. Equivalent or higher scores classify subjects as psychologically distressed. The subscales are also valid measures of severity of the emotional disorder. The Italian translation and validation of the scale was used [15]. In the present research the total score and the 2 sub-scales were chosen for statistical analysis.

*The Barthel Index, BI* [16]. The instrument is widely used to evaluate activities of daily living. The scale consists of 10 items that measure feeding, moving from wheelchair to bed and return, grooming, transferring to and from a toilet, bathing, walking on level surface, going up and down stairs, dressing, continence of bowels and bladder. The highest score is 100, i.e. total independence. Since motor fluctuations are common in PD, the best score for each item in the last week was taken.

### Intervention

First of all the diagnosis of idiopathic PD was confirmed by movement disorder specialists, according to the PD Brain Bank criteria [17]. All participants underwent Magnetic Resonance Image to exclude changes due to other diseases. The case history was aimed at excluding previous traumatic brain injuries and usage of dopaminergic blockers. Case history and clinical records were evaluated to exclude severe comorbidities, such as arthritis, cancer, arterial hypertension, small vessel and heart disease, and psychosis. A self administered questionnaire was proposed to all participants under investigators supervision.The duration of the interview was about three hours. The questionnaires were administered to the participants always in a different order. The MDS-UPDRS and the BI were assessed by a medical doctor with the assistance of a caregiver. Laborious procedures were avoided. Healthy volunteers were administered only the SOC scale.

### Analyses

PD and CG participants were compared on socio-demographic (i.e. Sex, Age and Education) and SOC by means of the Chi-square test for categorical variables and unpaired Student’s *t-*test for continuous variables. In the PD group, the Spearman’s correlation analysis was used to investigate the relationship between the SOC, and health-related and disease severity variables MDS-UPDRS, WHO-5, HADS and BI scores. The effect of SOC on disability, emotional distress and on well-being was investigated by means of log-linear regression. Mean ratios adjusted for sex, age, education, and MDS-UPDRS were estimated, along with 95% confidence intervals and p-values. Threshold for statistical significance was set at p < 0.05. The statistical software used for the statistical analyses STATA/SE V12 (StataCorp. 2011. *Stata Statistical Software: Release 12*. College Station, TX: StataCorp LP).

## Results

No statistical difference emerged between PD and CG on socio-demographic characteristics and SOC scores did not differ between the groups (Table 1). In PD the mean values of the 3 SOC factors were: SOC-C = 22.2 (SD = 4.1); SOC-Ma = 15.7 (SD = 3.1); SOC-Me = 15.8 (SD = 3.5). Corresponding values in CG were: SOC-C = 23.2 (SD = 4.1); SOC-Ma = 17.3 (SD = 2.8); SOC-Me = 15.7 (SD = 2.7).

A statistically significant positive correlation was found between SOC and QoL (WHO-5: corr = 0.40, p = 0.004), and a negative significant correlation between SOC and emotional distress (HADS Total: corr = −0.37, p = 0.008; particularly for anxiety HADS Anxiety: corr= −0.38, p = 0.006). As expected, the QoL was also highly correlated with anxiety and depression (HADS Total: corr = −0.65, p < 0.001; HADS Anxiety: corr = −0.51, p < 0.001; HADS Depression: corr = −0.57, p < 0.001). Full results are shown in Table 2.

**Table 2:** **Spearman's correlation analysis between socio-demographic, psychological and clinical characteristics in Parkinson's Disease subjects**

**Spearman's correlation analysis**	**SOC**	**UPDRS**	**WHO-5**	**HADS total**	**HADS - anxiety**	**HADS - depression**	**BARTHEL index**
**SOC**	1						
**UPDRS**	−0.0076	1					
	0.96						
**WHO-5**	0.399	−0.023	1				
	**0.004**	0.87					
**HADS total**	−0.371	0.2268	−0.6502	1			
	**0.008**	0.11	**< 0.001**				
**HADS - anxiety**	−0.3805	0.0803	−0.5114	0.8262	1		
	**0.006**	0.58	**< 0.001**	**< 0.001**			
**HADS - depression**	−0.2486	0.2988	−0.5719	0.7761	0.3761	1	
	0.08	**0.035**	**< 0.001**	**< 0.001**	**0.007**		
**BARTHEL index**	−0.1269	−0.1491	0.03	−0.1377	−0.0409	−0.2654	1
	0.38	0.30	0.84	0.34	0.78	0.06	

Statistical significant correlation: p < 0.05 (in bold).

From the log-linear regression analysis it was estimated that a one point increase in SOC has an average adjusted effect of −3% [95% CI: −5% to −1%, p = 0.01] on total emotional distress (model R-squared = 27%), while the effect on QoL is +2% [95% CI: +1% to +4%, p = 0.01] (model R-squared = 21%). No association was found between SOC and BI (model R-squared = 17%). These results are in line with those of the explorative correlation analysis reported above in Table 3.

**Table 3:** **Effect of Sense of Coherence (SOC) and on well-being (WHO-5: Quality of Life Questionnaire), anxiety and depression (HADS: Hospital Anxiety and Depression Scale) and disability (Barthel Index)**

	**HADS**			**WHO-5**			**Barthel index**		
	**Mean**	**95%** **CI**	**P-Value**	**Mean**	**95%** **CI**	**P-value**	**Mean**	**95%** **CI**	**P-value**
	**Ratio**			**Ratio**			**Ratio**		
**SOC**	0.97	[0.95; 0.99]	**0.01**	1.02	[1.01; 1.04]	**0.01**	1.00	[0.99 ; 1.00]	0.28
**Male**	0.76	[0.56; 1.04]	0.09	1.22	[0.96; 1.55]	0.10	1.11	[0.98; 1.25]	0.10
**Age**	1.00	[0.99; 1.02]	0.82	1.00	[0.99; 1.02]	0.48	1.00	[0.99; 1.01]	0.88
**Education**	0.97	[0.93; 1.00]	0.08	1.01	[0.98; 1.03]	0.70	1.01	[1.00; 1.03]	0.12
**UPDRS**	1.01	[0.99; 1.03]	0.27	0.99	[0.98; 1.01]	0.44	0.99	[0.99; 1.00]	0.10

Adjusting covariates: sex, age, education and disease severity (UPDRS: Unified Parkinson’s Disease Rating Scale).

*Log-transformation applied to original scores. 95% CI: 95% confidence interval. Statistical Significance: p < 0.05 (in bold).

## Discussion

According to Antonovsky’s salutogenic concept, a strong SOC is associated with physical and psychological health [3] and promotes behaviour by activating the appropriate resources. In Salutogenic Theory, the generalized resource deficits have a negative impact. On the other hand, the generalized resistance resources help a person to cope with the illness by avoiding or contrasting a range of stressors. Therefore, the perceived health should improve and this will have a positive influence on the physical component of QoL [18]. According to Antonovsky, SOC develops during childhood and early adulthood and stabilize around the age of 30, although Empirical values show an increase in normal aging [10]. A highly significant inverse association was described between baseline SOC and depressive symptoms at 1 year and at 9 years, supporting the hypothesis the low SOC is a predictor of depression both in short and long term [19]. On the other hand, a high SOC may have a protective function. In the aging a positive relationship between SOC and well-being is found in other studies [20]. Elderly individuals with a strong SOC are protected against depression with higher levels of satisfaction with their life.

On these premises much research is warranted in the field of PD, which is a progressive, degenerative and disabling disease characterized by motor, autonomic, cognitive and affective signs. Moreover, personality changes have been reported. PD patients are more suspicious and cautious than either healthy age-matched individuals or patients suffering from other chronic diseases. With relative uniformity, they have been described as stoic, serious, rigidly moral, non-flexible, non-impulsive, and with less novelty-seeking behaviour [21–26]. Personality is influenced by the decrease of the dopaminergic turn-over, which can be responsible for depression and reduced executive functions. As we already reported [4], a pronounced decrease in the SOC scale (p < 0.0001) occurs in PD subjects over time. Furthermore, PD subjects show reduced active coping strategies and lower well-being scores. The coping mechanisms in PD and non-neurological patients are different. PD subjects are characterized by lower SOC and higher scores of depression. However, SOC correlates with mood, coping, well-being and HR-QoL [5].

Our results confirm the positive association between SOC and QoL and the negative association with emotional distress, although the model R2 were not fully satisfying. Our results are similar to other findings [5], but we failed to find a lower SOC in PD compared to CG (p = 0.08). The result appears unexpected because SOC in PD is lower than SOC in other non-neurological diseases [5]. A possible interpretation of this - somehow contradictory - finding is linked to the fortuitous lowering of the SOC in the general population due to the serious economical and political crisis of the last year. Nowadays, people are generally sceptic about their future and the suicidal rate is increasing. If we compare our SOC values with normative data drawn from an Italian population in 2010 [10], we can see that our median value is 54 instead of the expected value of 60. Therefore, the so-called ‘healthy volunteers’ are plagued by worries and problems which challenge their SOC. Other explanations, such as the slightly higher education level of CG should be considered.

Our observations are in line with other results in PD [5] about the lack of correlation with disability and in ostheoarthritis, where the same association could not be found [27]. SOC is not the only personality’s traits to be uncorrelated with disability, infact the same result was found for Dispositional Optimism [28,29]. At this time the only personality’s trait which could predict disability is the internal Locus of Control [28]. Only in the referred study, greater internal Locus of Control was associated with less PD disability.

Our study in part confirms the Salutogenetic Theory because a strong SOC positively influences psychosocial health. On the other hand, this effect is not so evident when one considers physical health. The same conclusions are reported in other studies [5,27,30]. SOC seems to be comparatively stable, but not as stable as Antonovsky initially assumed, and factorial structure of SOC seems to be rather multidimensional [18]. According to another study [31] the SOC scale requires further psychometric studies. It is still unclear about the number of factors of the model grouping the items.

In spite of some limitations in the Salutogenic Theory, SOC scale is a world-widely used instrument in medicine as well as other research areas. SOC permits to ascertain individual’s orientation and internal resources for coping strategies. This possibility is certainly useful in the PD care.

Finally, we acknowledge the limitations of our study. The participants of the research are not representative of the typical PD phenotype. Rather, they are subjects in the early stages and the median value of MDS-UPDRS is 40. Indeed, higher values of disability would not allow the patients’ rehabilitation in a DH protocol. Secondly, many testing procedures like SOC require hand-writing, which is severely impaired in advanced stages of the disease. Another limitation of the study is the lack of evaluation of depression and QoL in the CG. When the study was designed we did not expect to hypothesize a clear decrease of SOC in the healthy volunteers.

## Conclusion

In conclusion, SOC is predictive of QoL and emotional distress in PD. A decreased SOC could not counteract negative life events. The more negative life events, the more decrease in SOC. Interventions might play a role for sustain SOC under stressful conditions [32]. Further studies are warranted to verify whether SOC at baseline is predictive of rehabilitation gain. Psychological interventions may reduce the use of escape-avoidance coping [33], especially in young PD patients who generally present with a reduced.

QoL compared to older-onset patients [34]. Reinforcing SOC in a rehabilitation program may also control depression, which is known to reduce QoL by 40% in PD patients [35].

## Abbreviations

MMSE: Mini mental state examination
CIRS: Cumulative illness rating scale
MDS-UPDRS: Movement disorder society-sponsored revision of the unified Parkinson's disease rating scale
SOC: Sense of coherence
HADS: Hospital anxiety and depression scale
BI: The barthel index
